# Injury severity and pattern in motor vehicle accident victims experiencing seatback offset or wreckage by unrestrained objects in the car compartment or boot

**DOI:** 10.1186/1757-7241-20-S1-O7

**Published:** 2012-03-22

**Authors:** T Staff, T Eken, TB Hansen, PA Steen, S Søvik

**Affiliations:** 1 Department of Research, Norwegian Air Ambulance Foundation, Drøbak, Norway; 2 Department of Anaesthesiology, Oslo University Hospital, Ullevål, Norway; 3 Division of Pre-hospital Medicine, Oslo University Hospital, Ullevål, Norway; 4 Institute of Clinical Medicine, University of Oslo, and Division of Pre-hospital Medicine, Oslo University Hospital, Ullevål, Norway; 5 Department of Anaesthesiology, Akershus University Hospital, Lørenskog, Norway

## Background

The European UN ECE17 regulation states that car backseats should sustain hits from 36 kg luggage in 20 to 28 g. This weight seems unrealistically low; still during crash tests such loads wrecked seatbacks and injured rear seat test dummies.

## Methods

In car crashes with suspected severe person injury, dedicated paramedics documented location and movement of unrestrained objects in the vehicle and seatback offset or wreckage. Patient injury pattern (AIS codes) and severity (NISS scores) were determined, and potential relation to unrestrained objects was explored.

## Results

190 accidents involving 338 motor vehicles (618 persons, 65% men) were included. 133 vehicles had potentially harmful unrestrained objects. Seatback offset caused by moving unrestrained objects was reported for 45 persons. A subgroup analysis was performed of 17 persons where death or severe injury seemed strongly associated with seatback offset or wreckage caused by moving unrestrained objects. 10/17 persons were women, median age was 40 years (IQR 25.5–54). 9/17 were passengers, 10/17 used a seatbelt. 15/17 collisions were frontal, median velocity range was 65–75 km/h. Seatback offset was mainly associated with luggage weighing >34 kg (11/17, 65%). Median seatback offset was 16 cm (range 8–22) for belted persons and 25 cm (range 10–80) for unbelted persons. 10/17 died at the site of accident. Median maximum AIS score was 5 (IQR 4–6). Median NISS was 45.5 (range 10–75) for belted persons and 75 (range 21–75) for unbelted persons. Seatback offset or wreckage was associated with severe injuries in the thoracic, abdominal and pelvic region. Unbelted persons had more frequent and more severe extremity injuries and more severe injuries to the cervical spine.

## Conclusions

Unrestrained objects in crashing cars may wreck seatbacks and cause serious injury. There is a need for a marking system for seatback weight resistance and improved use of cargo barriers, nets and straps.

